# Genome-Wide Association Study of Campylobacter*-*Positive Diarrhea Identifies Genes Involved in Toxin Processing and Inflammatory Response

**DOI:** 10.1128/mbio.00556-22

**Published:** 2022-04-14

**Authors:** Rebecca M. Munday, Rashidul Haque, Ning-Jiun Jan, Genevieve L. Wojcik, Chelsea Marie, Dylan Duchen, Alexander J. Mentzer, Uma Nayak, Poonum Korpe, Beth D. Kirkpatrick, William A. Petri, Priya Duggal

**Affiliations:** a Department of Genetic Medicine, Johns Hopkins School of Medicine, Baltimore, Maryland, USA; b International Centre for Diarrhoeal Disease Research, Dhaka, Bangladesh; c Department of Medicine, Infectious Diseases and International Health, University of Virginia School of Medicine, Charlottesville, Virginia, USA; d Department of Epidemiology, Johns Hopkins Bloomberg School of Public Health, Baltimore, Maryland, USA; e The Wellcome Centre for Human Genetics, University of Oxford, Oxford, Oxfordshire, UK; f Center for Public Health Genomics and Department of Public Health Sciences, University of Virginia School of Medicine, Charlottesville, Virginia, USA; g University of Vermont College of Medicine and Vaccine Testing Center, Burlington, Vermont, USA; Emory University School of Medicine

**Keywords:** Campylobacter, diarrhea, genetics

## Abstract

Diarrhea is responsible for the deaths of more than 500,000 children each year, many of whom reside in low-to-middle-income countries (LMICs). Additionally, children with multiple diarrheal infections early in life have increased growth stunting and malnutrition and decreased vaccine efficacy. Two bacteria that contribute to the burden of diarrhea are Campylobacter jejuni and Campylobacter coli, both are endemic in Bangladesh. However, not all children that are exposed to these pathogens, including Campylobacter, will experience diarrhea. We hypothesized that host genetics may influence susceptibility to Campylobacter infections and performed a genome-wide association study in 534 children from two independent birth cohorts in Dhaka, Bangladesh. Infants were monitored for diarrhea for the first 2 years of life and only defined as controls if all diarrheal samples in the first year were negative for Campylobacter jejuni*/*C. coli. Each cohort was analyzed separately under an additive model and adjusted for length-for-age z-scores at birth and 12 months, sex, water treatment, and ancestry. In a fixed effect inverse-variance weighted meta-analysis of these two cohorts, we identified a genome-wide significant region on chromosome 8 in intron 4 of the rho guanine nucleotide exchange factor 10 gene (*ARHGEF10*). Individuals with the G allele (rs13281104) had a 2-fold lower risk of having a Campylobacter-associated diarrheal episode than individuals with the A allele (OR 0.41, 95% CI 0.29 to 0.58, *P* = 3.6 × 10^−7^). This SNP is associated with decreased expression of the neighboring gene, *CLN8*, which may be involved in the transport of the cytolethal distending toxin produced by Campylobacter.

## INTRODUCTION

Campylobacter species are the leading cause of bacterial gastroenteritis in humans worldwide, the most common being C. jejuni and C. coli (https://www.who.int/news-room/fact-sheets/detail/campylobacter). WHO estimated more than 166 million illnesses and 3,733,822 disability-adjusted life years (DALYs) from Campylobacter globally in 2010 (1). The annual incidence of infection varies regionally from 14.3/100,000 people in the United States (2) to 1,512/100,000 people in Japan (2) to endemicity in low-resource countries (3–6). While there are undoubtedly differences in surveillance and detection methods, a larger factor in this disparity is that the natural reservoirs of Campylobacter species differ around the world (2). In wealthier countries, the main sources of Campylobacter are poultry and cattle (7). Human infections are sporadic and usually linked to the ingestion of contaminated food. Developed countries see a rise in Campylobacter infections in the warmer months (7) while developing countries often show no seasonality (8). In low-to-middle-income countries (LMICs) the bacteria are ubiquitous in the environment, caused in part by asymptomatic shedding (3, 5, 8) and the bacterium’s ability to survive for days in feces, soil, and water (9). A birth cohort study of 8 low-resource settings looked at both diarrheal and surveillance (nondiarrheal) stool samples and found that about 85% of children had at least one Campylobacter-positive stool sample by 1 year of age (5). The two most common pathogenic Campylobacter species, C. jejuni, and C. coli are also highly similar and as many tests do not differentiate between the two species, they are often reported together as C. jejuni*/*C. coli. In a separate birth cohort from Dhaka, Bangladesh, C. jejuni*/*C. coli were implicated in approximately 11.3% of all diarrheal episodes, second only to enteroaggregative Escherichia coli (10).

Campylobacter infection includes fever, nausea, and bloody diarrhea lasting 3 to 6 days (https://www.who.int/news-room/fact-sheets/detail/campylobacter). Although adults and children are infected with Campylobacter, young children have visible long-term sequelae from Campylobacter diarrheal and nondiarrheal infections, including growth stunting (5, 11). In LMICs, a high burden of Campylobacter has been associated with decreased linear growth at 24 months of age and an increase in systemic inflammation (5). C. jejuni has also been identified as an important factor in environmental enteric dysfunction, in which children suffer from malabsorption, cognitive deficits, and increased risk of death (12).

Risk factors for Campylobacter infection include malnutrition, lack of access to clean water, and poor sanitation, although direct contact with animals may also play a role in transmission (5, 13). Many global health initiatives have focused on improving these factors, but enteric infections persist (14). Thus far, attempts to develop a Campylobacter vaccine have been complicated by the association between Campylobacter infection and Guillain-Barré Syndrome (GBS), a debilitating condition in which the body’s immune system attacks the peripheral nerves. GBS develops in approximately 0.1% of individuals postinfection (15). Vaccine efforts have also been hindered by an incomplete understanding of the intricacies of Campylobacter pathogenesis *in vivo*, largely due to insufficient animal models that do not recapitulate the human phenotype (16). Identification of host susceptibility factors could aid vaccine development by elucidating intracellular processes.

Genetic variants influencing host susceptibility to infection have been found for many infectious agents, including viruses, bacteria, and protozoa (17, 18). Here, we aimed to identify genetic variants associated with host susceptibility to Campylobacter infection using two well-characterized birth cohorts: the Performance of Rotavirus and Oral Polio Vaccines in Developing Countries (PROVIDE) Study (19) and the Cryptosporidiosis Birth Cohort (CBC) (20).

## RESULTS

### Descriptive statistics.

We evaluated diarrhea-associated Campylobacter infections in both PROVIDE and CBC. In the PROVIDE Study, there were 218 children with at least one diarrheal sample positive for C. jejuni*/*C. coli (218/385) in the first year of life. Of these 218 cases, 89 were female and 129 were male. The average age at which children had their first Campylobacter-positive diarrhea was 221 days, and this was not associated with sex (*P* = 0.70). Among cases, 45.4% of children (99/218) had more than one diarrhea-associated Campylobacter infection in the first year of life. In CBC there were 91 children with at least one diarrheal sample positive for C. jejuni*/*C. coli (91/149) in the first year of life. Of these 91 cases, 52 were female and 39 were male. The average age at which these children had their first Campylobacter*-*positive diarrhea was 212 days, and there was no association with sex (*P* = 0.81). Among cases, 43.9% of children (40/91) had more than one diarrhea-associated Campylobacter infection in the first year of life. Controls in PROVIDE averaged higher LAZ at 12 months and were more likely to live in households that practiced routine water treatment (Table 1). No risk factors were significantly associated with outcome in CBC.

**TABLE 1:** Individual cohort demographics

	PROVIDE			CBC		
Covariate	Cases N = 218	Controls N = 167	*P* value^*a*^	Cases N = 91	Controls N = 58	*P* value
Sex^*b*^ (% female)	41	50	0.10	57	55	0.95
Exclusive breastfeeding (mean number days)	113	119	0.38	118	119	0.99
Toilet (% improved)	14	15	0.85	31	29	0.99
LAZ^*a*^ birth^*b*^ (mean)	−0.97	−0.89	0.36	−0.92	−0.77	0.32
LAZ 12 mo^*b*^ (mean)	−1.6	−1.3	0.015	−1.3	−1.3	0.95
WAZ^*a*^ 12 mo (mean)	−1.3	−1.1	0.072	−1.1	−0.92	0.29
Water treatment^*b*^ (% treating)	54	69	0.0032	74	83	0.27

a LAZ, length-for-age Z-score; WAZ, weight-for-age Z-score; *P* is the *P* value from a chi-square test (categorical data) or *t* test (continuous data).

b Covariate included in the final regression model.

### Association analysis.

**FIG 1:**
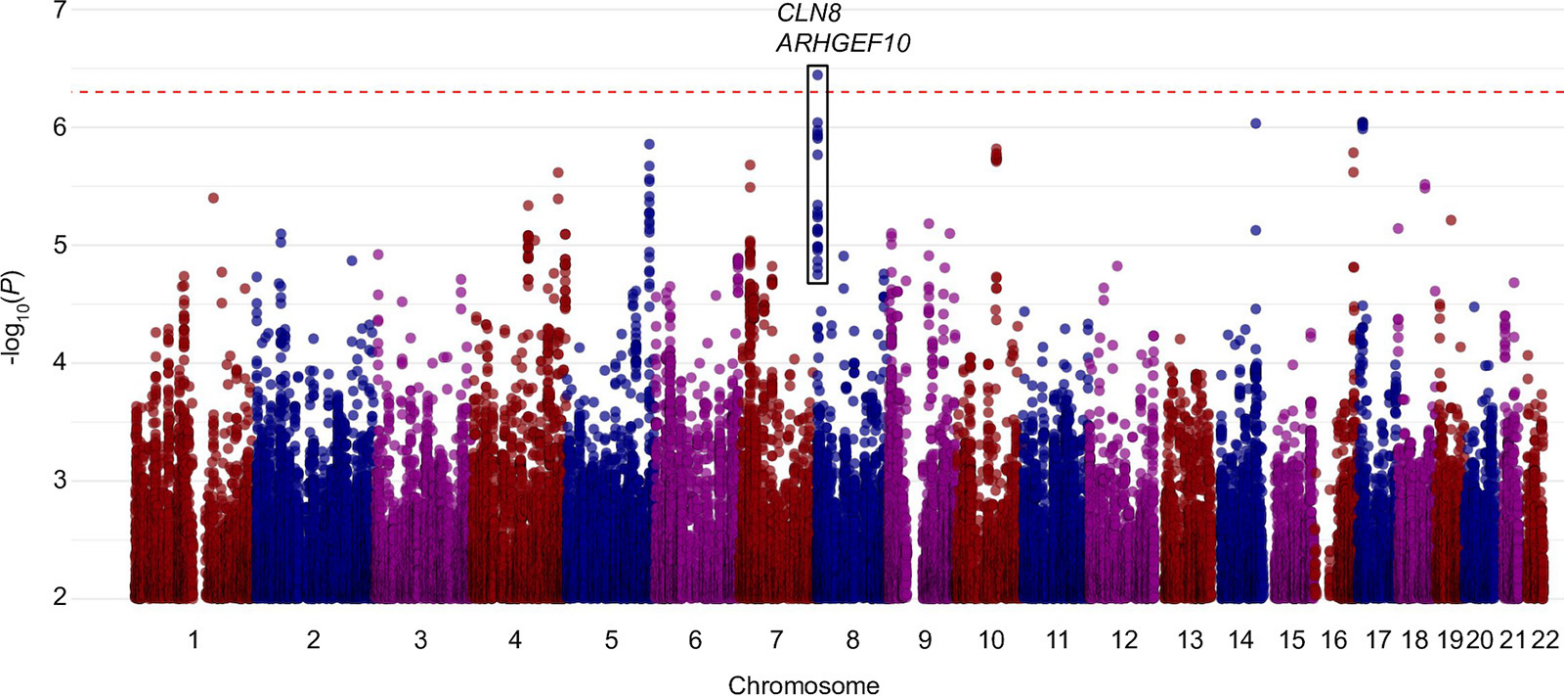
Meta-analysis of Campylobacter-associated diarrhea revealed a significant locus on chromosome 8. Each dot represents a single variant, sorted by chromosomal location along the *x*-axis. The *y* axis is the −log_10_ *P* value in the meta-analysis of the two cohorts, PROVIDE and CBC. Each cohort was adjusted for sex, LAZ at birth, LAZ at 12 months, water treatment, and the top principal component. The red line indicates genome-wide significance (5 × 10^−7^).

In the meta-analysis of study-specific GWAS, we identified a genome-wide significant locus on chromosome 8 associated with protection from Campylobacter-associated diarrhea (Fig. 1), located in intron 4 of the Rho Guanine Nucleotide Exchange Factor 10 gene (*ARHGEF10*) (Table 2). The highest-scoring SNP, rs13281104, had an allelic odds ratio (OR) of 0.41 (95% confidence interval (CI) 0.29 to 0.58, *P* = 3.6 × 10^−7^, minor allele frequency (MAF)_cases_ = 0.12, MAF_controls_ = 0.24). Children with one copy of the G allele were 2.4 times less likely to have a diarrhea-associated Campylobacter infection compared to children without the G allele. The odds ratios and *P* values were consistent in both cohorts (Table 2).

**TABLE 2:** Highest-Scoring SNPs

					PROVIDE		CBC		Meta-Analysis				
rsID	Chr:Pos^*a*^	A0^*a*^	A1^*a*^	Pop MAF^*a*^	OR (95% CI)	*P* value^*b*^	OR (95% CI)	*P* value^*b*^	Case MAF	Control MAF	OR (95% CI)	*P* value^*c*^	*P_het_* ^*a*^
rs13281104	8:1811923	A^*d*^	G	0.16	0.45 (0.30, 0.67)	9.6 × 10^−5^	0.35 (0.19, 0.65)	8.6 × 10^−4^	0.12	0.24	0.41 (0.29, 0.58)	3.6 × 10^−7^	0.52
rs13272734	8:1817756	C^*d*^	G	0.17	0.46 (0.31, 0.69)	1.6 × 10^−4^	0.37 (0.20, 0.68)	1.3 × 10^−3^	0.12	0.24	0.43 (0.31, 0.60)	9.1 × 10^−7^	0.54
rs7003839	8:1817314	G^*d*^	C	0.17	0.46 (0.31, 0.69)	1.7 × 10^−4^	0.37 (0.20, 0.69)	1.6 × 10^−3^	0.12	0.24	0.44 (0.31, 0.61)	1.1 × 10^−6^	0.56
rs13277141	8:1815147	G^*d*^	C	0.18	0.46 (0.31, 0.68)	8.5 × 10^−5^	0.41 (0.22, 0.75)	3.9 × 10^−3^	0.13	0.25	0.45 (0.32, 0.62)	1.1 × 10^−6^	0.75
rs13252399	8:1815012	T^*d*^	A	0.17	0.47 (0.31, 0.70)	1.9 × 10^−4^	0.37 (0.20, 0.68)	1.5 × 10^−3^	0.12	0.24	0.44 (0.31, 0.61)	1.2 × 10^−6^	0.54
rs13267804	8:1813692	C^*d*^	T	0.17	0.47 (0.31, 0.69)	1.7 × 10^−4^	0.38 (0.21, 0.70)	1.8 × 10^−3^	0.12	0.24	0.44 (0.31, 0.61)	1.2 × 10^−6^	0.58
rs17829629^*e*^	8:1817961	C	A^*d*^	0.15	0.47 (0.31, 0.70)	1.9 × 10^−4^	0.38 (0.21, 0.71)	2.3 × 10^−3^	0.12	0.23	0.44 (0.31, 0.61)	1.7 × 10^−6^	0.60

a Chr, chromosome; Pos, position; A0, reference allele; A1, tested allele; Pop MAF, minor allele frequency reported in 1000 Genomes for the Bengali in Bangladesh population; OR, odds ratio; CI, confidence interval; *P_het_*, *P* value of heterogeneity from Cochran’s Q.

b *P* value from frequentist association test.

c *P* value from the meta-analysis.

d Ancestral allele.

e Genotyped SNP.

### Functional assessment.

**FIG 2:**
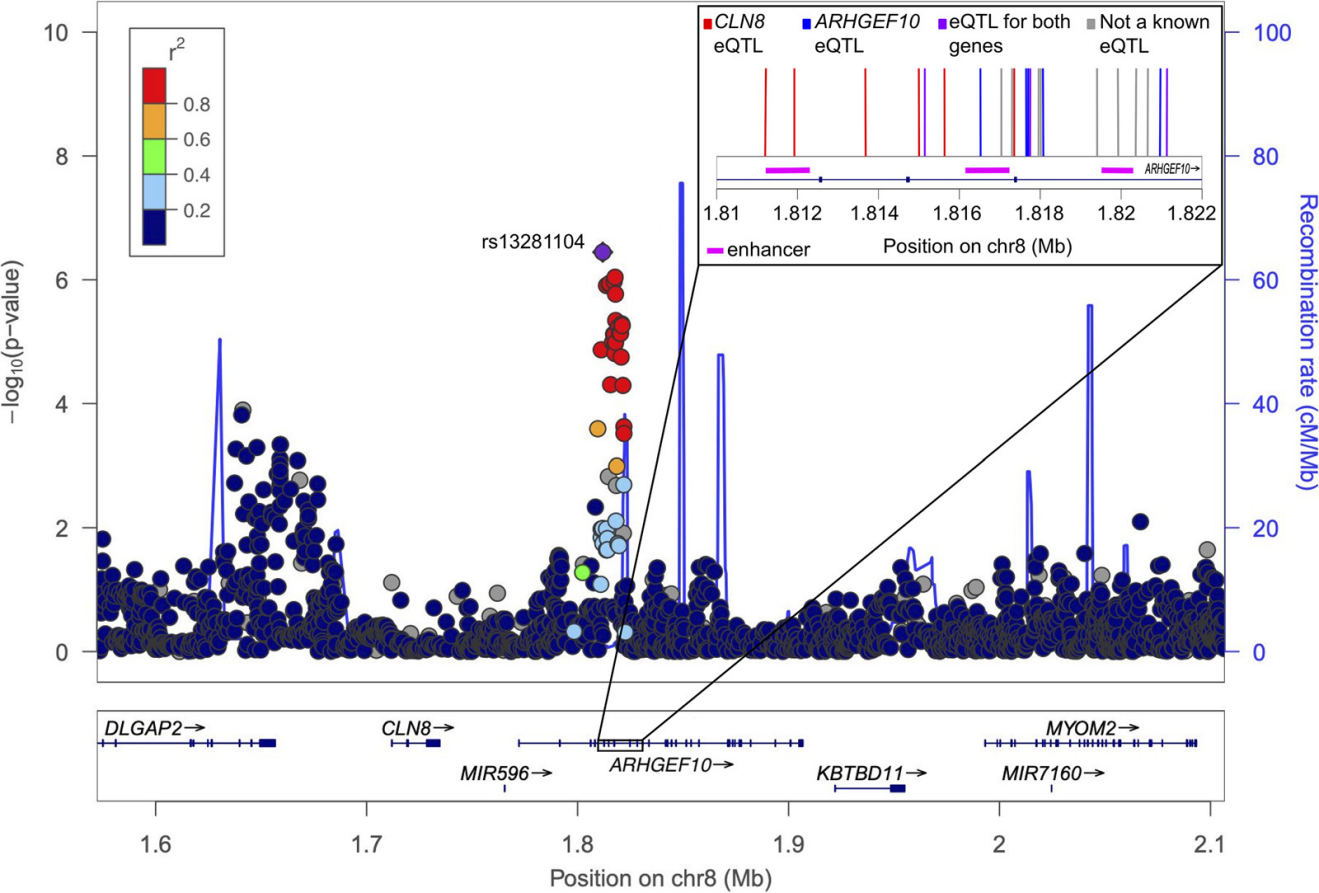
Genomic context. Each dot represents a single variant, ordered by position on chromosome 8 along the *x*-axis. The *y* axis on the left shows the -log_10_ *P* value from the meta-analysis, while the *y* axis on the right indicates the recombination rate in centimorgans per megabase. The colors indicate linkage disequilibrium (r^2^) between each variant and the highest-scoring SNP (rs13281104, shown in purple). The zoomed-in portion shows exons 5 to 7 of *ARHGEF10* and the 22 variants in the LD block with *P* < 5 × 10^−5^ in the meta-analysis, colored by effect listed in GTEx. Red lines are *CLN8* eQTLs in whole blood, blue lines are *ARHGEF10* eQTLs in the brain, purple lines are eQTLs for both genes (*CLN8* in blood and *ARHGEF10* in the brain), and gray lines are not identified as significant eQTLs in GTEx. The pink bars along the bottom indicate the approximate locations of enhancers listed in GeneCards.

The highest-scoring SNP, rs13281104, is in a linkage disequilibrium (LD) block (r^2^ > 0.8) spanning approximately 10 kb on chromosome 8, including variants within introns 4, 5, 6, and 7 of *ARHGEF10* (Fig. 2). Our search for functional variants in this region revealed 3 enhancers that share the same 4 gene targets: 2 long noncoding RNAs (*lnc-CLN8-4* and *lnc-ERICH1-8*) and 2 protein-coding genes (*ARHGEF10* and *AC019257.8*). Two of these enhancers (GH08J001867 and GH08J001871) are active in immune cell types (CD14^+^ monocytes and natural killer cells (21); common myeloid progenitor CD4^+^, M0 macrophage, CD14^+^CD16^−^ monocytes (22)) and 1 enhancer (GH08J001863) is repressed in some immune cells (B cells, spleen, and CD4^+^CD25^+^ ab Treg (22)).

In addition to rs13281104, there were 21 other variants in LD (r^2^ > 0.8) that had *P* < 5 × 10^−5^ in the meta-analysis. However, none of these 22 variants were identified in GTEx as significant eQTLs for *ARHGEF10* in the sigmoid colon, transverse colon, small intestine, spleen, or whole blood. Eight of the variants (8/22) were significant eQTLs for *ARHGEF10* in the cerebellar hemisphere of the brain (normalized effect sizes ≤ −0.45, *P* < 3.6 × 10^−5^). Nine (9/22) were significant eQTLs for the neighboring gene, *CLN8*, in whole blood (normalized effect sizes ≤−0.12, *P* < 6.6 × 10^−5^). None were significant eQTLs for *CLN8* in the sigmoid colon, transverse colon, small intestine, or spleen. Overall, 14 of the highest-scoring variants (14/22) were significantly associated with decreased gene expression of *ARHGEF10*, *CLN8*, or both (Fig. 2 inset).

We included rs13281104 in the model as a covariate to determine if the SNP accounted for the signal for this region (Chr 8:1810000-1822000), and when included in the model no further associations were observed for the region (Fig. S2). This suggests that the putative functional SNP is in this region and has high linkage disequilibrium with rs13281104. Conditioning on the highest-scoring SNP, rs13281104, also revealed additional areas of interest as 3 new loci reached genome-wide significance (*P* < 5 × 10^−7^). The three regions were: a single variant in the potassium channel gene *KCNK13*, on chromosome 14 (rs34114368, OR = 0.47, *P* = 2.6 × 10^−7^), several intergenic variants on chromosome 16 (top variant rs11643120, OR = 2.2, *P* = 3.5 × 10^−7^), and several variants on chromosome 17 within the SNARE complex gene syntaxin 8 (*STX8*). The highest-scoring variant in *STX8* was rs73973765 (OR = 0.35, *P* = 2.5 × 10^−7^).

10.1128/mbio.00556-22.2 FIG S2 Manhattan plot of conditional meta-analysis. Download FIG S2, PDF file, 0.3 MB. Copyright © 2022 Munday et al. 2022 Munday et al. https://creativecommons.org/licenses/by/4.0/ This content is distributed under the terms of the Creative Commons Attribution 4.0 International license.

Given that these *STX8* variants did not reach significance in the original meta-analysis, we wondered whether they were indeed independent of *ARHGEF10* and just affected by sample size or if there may be an interaction between the two loci. We looked at the odds ratios for infants carrying protective alleles in only one gene (*ARHGEF10* or *STX8*) as well as those carrying protective alleles in both genes (Table 3). While infants carrying protective alleles in both genes did have a slightly lower odds ratio than would be expected for independent SNPs, it was not statistically significant and may simply reflect the small sample size.

**TABLE 3:** Assessment of interaction

	Control	Case	OR	95% CI
No protective alleles (reference)^*a*^	84	199		
Protective allele(s) in *ARHGEF10* only^*b*^	67	59	0.37	(0.24, 0.57)
Protective allele(s) in *STX8* only^*b*^	43	37	0.36	(0.22, 0.60)
Protective allele(s) in both genes^*c*^	25	6	0.10	(0.04, 0.26)

a Infants carrying neither rs13281104 nor rs73973765. Because rs73973765 was an imputed SNP, hard genotype calls were utilized for this table, with a cutoff of 0.9. Infants with probability weights <0.9 for all genotypes at this locus were excluded from this analysis.

b Infants carrying either one or two copies of the highest-scoring SNP for that gene.

c Infants carrying at least one copy of each highest-scoring SNP (rs13281104 and rs73973765).

**FIG 3:**
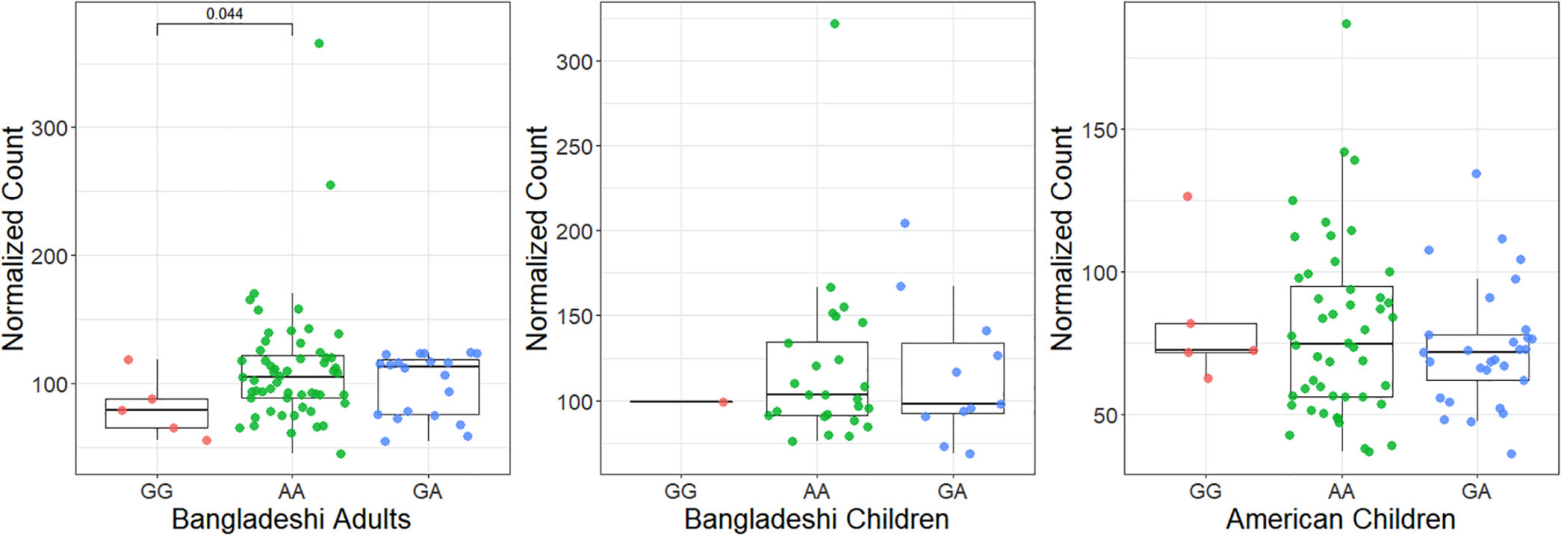
RNA-Seq Results for rs13281104. In each panel, GG genotype counts are in red, AA genotype counts are in green, and GA genotype counts are in blue. A significant difference in counts (*P* < 0.05) was found when comparing between those with GG and AA phenotypes in the Bangladeshi Adult cohort.

In the RNA-Seq experiment, we found that Bangladeshi adults carrying two copies of the G allele (rs13281104) had significantly less expression of *ARHGEF10* in the small intestine compared to individuals with two copies of the A allele (Fig. 3). There were not enough Bangladeshi children with the GG genotype to make the same comparison. No significant differences were observed in the expression of *ARHGEF10* by genotype in American children. We also compared the expression of *CLN8*, *KCNK13*, and *STX8* and found no differences in expression by genotype in any of the 3 cohorts.

## DISCUSSION

Through a GWAS of diarrhea-associated Campylobacter infection in Bangladeshi infants, we identified genetic variants in introns of *ARHGEF10* associated with protection from Campylobacter*-*positive diarrhea, potentially through decreased expression of *CLN8*. Our region of interest includes 3 enhancers, 2 of which are active in monocytes and 1 which is repressed in regulatory T cells. The enhancers have multiple targets, including *ARHGEF10* and a long noncoding RNA known as lnc-CLN8-4. Variants in enhancers can impact gene expression by altering transcription factor binding and many previous studies have identified SNPs in enhancers as disease risk loci (23, 24). Given that some of our top variants were eQTLs for both *ARHGEF10* and *CLN8*, either gene could be responsible for the observed phenotype.

*CLN8* encodes a transmembrane protein that localizes primarily to the endoplasmic reticulum and functions as a transporter between the ER and the ER-Golgi intermediate compartment (ERGIC) (25). Mutations in this gene are responsible for two neurodegenerative diseases: neuronal ceroid lipofuscinosis-8 (OMIM 600143) and Northern epilepsy (OMIM 610003). *CLN8* has been linked to ceramide synthesis, lipid homeostasis, and vesicular transport (26, 27). Prior studies have shown host cell lipid composition to be important both for pathogenic invasion and innate immune response (28, 29). One of the identified protein partners of CLN8 is the SNARE complex protein STX8 (27), and the most significant variant in our conditional analysis was in the *STX8* gene. Through this previously identified protein interaction with STX8 (27), CLN8 may be involved in the trafficking of the cytolethal-distending toxin secreted by Campylobacter species. This toxin is transported from the plasma membrane to the Golgi apparatus, then the endoplasmic reticulum, and eventually the nucleus, where it damages DNA and causes cell death (30). Reduction of *CLN8* expression might impair endosome trafficking and thus reduce the lethality of the toxin, leading to a decrease in the incidence of Campylobacter-associated diarrhea. The variants that were identified as eQTLs for *CLN8* were all associated with its decreased expression in whole blood. There were other nearby SNPs (not shown) in the GTEx database that were identified as eQTLs for *CLN8* in the transverse colon, but they are not prevalent in the Bengali population. The reverse may be also true where there may be SNPs that affect gene expression in this population that is not observed in the largely European ancestry individuals that comprise the GTEx data set. If the reduced expression of *CLN8* is responsible for protection from Campylobacter-associated diarrhea, we would expect that mutations leading to an increase in *CLN8* expression would result in increased susceptibility to Campylobacter-associated diarrhea.

*ARHGEF10* encodes a rho guanine nucleotide exchange factor, responsible for facilitating the exchange of GDP for GTP, activating small rho GTPases (31). One mutation, downstream of our region of interest and not in LD, has been implicated in slowed nerve conduction velocity (OMIM 608236). Other downstream SNPs in *ARHGEF10* are associated with the risk of ischemic stroke in East Asian populations (32, 33). Rho GEFs can have several targets and the most well-studied for *ARHGEF10* are RhoA, RhoB, and RhoC (34, 35). These GTPases share 88% homology but have different functions in the cell (36). RhoA is involved in stress fiber formation and actomyosin contractility (34). It has also been implicated in intestinal inflammation via the Rho/ROCK signaling pathway (37). RhoB is localized to endosomes and has been shown to play a role in the signaling of apoptosis as a stress response (38). RhoB is also part of the signaling cascade that leads to inflammation in response to bacterial lipopolysaccharide (39). RhoC is involved in cell migration and has been studied in many cancer types (35). One of the ways that RhoC contributes to metastasis is through the weakening of adherens junctions (40).

Reduced expression of *ARHGEF10* in the small intestine of individuals with the G allele aligns with the eQTLs in the brain found in the GTEx database. This reduced expression may lead to a reduction in the inflammatory response, corresponding to a reduced likelihood of diarrhea.

Previous studies looking for associations between host genetic variation and Campylobacter susceptibility have focused on candidate genes such as those encoding lipopolysaccharide receptors and inflammatory cytokines, and no such associations have been found (41, 42). By utilizing a genome-wide approach we were able to identify a novel locus, whose precise role in Campylobacter pathogenesis is yet to be determined. As we learn more about the intracellular signaling that takes place during Campylobacter infection, we will be better equipped to develop effective vaccines. Identification of any gene that is protective from Campylobacter is critical for our understanding of innate immunity.

Our analysis focused on diarrheal samples and thus our associations are limited to diarrhea-associated Campylobacter infections and cannot readily be extended to overall resistance to infection or asymptomatic Campylobacter infections. Additionally, we did not have data on Guillain-Barré Syndrome in this population and therefore could not test any hypotheses related to its development after Campylobacter infection. This is the first genome-wide study in humans and would benefit from validation with future studies. The most comprehensive genetic work to date has been done in chickens, which do not generally experience diarrhea upon colonization and are thus not directly comparable to our study.

These children experience multiple enteric assaults and harbor multiple enteric pathogens. However, our genetic findings are distinct from previous genetic work that identified loci for both Entamoeba histolytica (18) and *Cryptosporidium* (43), which suggests that this association is unique to Campylobacter. In conclusion, we demonstrate that there is an association between rs13281104, an eQTL for *CLN8* in whole blood, and *ARHGEF10* in the small intestine, and decreased incidence of Campylobacter-positive diarrhea in the first year of life in infants in Dhaka, Bangladesh.

## MATERIALS AND METHODS

### Ethics statement.

The study protocol was approved by the Institutional Review Boards of the International Center for Diarrheal Disease Research, Bangladesh, University of Virginia, and Johns Hopkins Bloomberg School of Public Health. The parents or guardians of all individuals provided informed written consent.

### PROVIDE study design.

The “Performance of Rotavirus and Oral Polio Vaccines in Developing Countries” (PROVIDE) Study (19) is a randomized, controlled clinical trial evaluating the efficacy of a modified vaccine schedule, namely, a delayed-dose oral rotavirus vaccine and replacement of one dose of the oral polio vaccine with an injectable dose. The goal was to understand factors contributing to reduced vaccine efficacy in areas of poor sanitation, overcrowding, and poverty. A birth cohort of 700 infants from the Mirpur area of Dhaka, Bangladesh was established, following infants for the first 2 years of life. Bi-weekly diarrhea surveillance was conducted, and samples were tested for a variety of pathogens via quantitative real-time PCR (qRT-PCR) on a TaqMan Array Card (44). Other information collected in the study included length-for-age Z-scores (LAZ), weight-for-age Z-scores (WAZ), number of days of exclusive breastfeeding, maternal health information, and socioeconomic data.

### PROVIDE case definition.

For this study, cases were defined as infants (under 1-year-old) with at least one diarrheal sample testing positive for Campylobacter jejuni*/*C. coli. The TaqMan Array Card does not differentiate between Campylobacter jejuni and C. coli, so these are reported as a single result (C. jejuni*/*C. coli). Controls were defined as infants (under 1-year-old) with all diarrheal samples testing negative for these Campylobacter pathogens.

### CBC study design.

The Cryptosporidiosis Birth Cohort (20) was designed to evaluate the incidence of cryptosporidiosis and its effect on growth in childhood. Birth cohorts were established in two sites in Bangladesh: Mirpur and Mirzapur. Infants were followed for at least the first 2 years of life, with bi-weekly diarrhea surveillance conducted at their homes. A subset of those in the Mirpur area (*n* = 220) had their diarrheal samples tested for a variety of pathogens using qRT-PCR on a TaqMan Array Card (44). Other data collected included maternal health information, household income, length-for-age Z-scores, weight-for-age Z-scores, and the number of days of exclusive breastfeeding.

### CBC case definition.

Cases were defined as infants (under 1-year-old) with at least one diarrheal sample testing positive for C. jejuni*/*C. coli. Controls were defined as infants (under 1-year-old) with all diarrheal samples testing negative for these Campylobacter pathogens.

### Genotyping, quality control, and imputation.

From the PROVIDE cohort, 541 infants were genotyped using Illumina’s Expanded Multi-Ethnic Genotyping Array (MEGA-EX). From the CBC, 630 infants were genotyped using Illumina’s Infinium Multiethnic Global Array (MEGA). Illumina’s GenomeStudio was used for genotyping quality control, and we removed 676,854 markers (PROVIDE) and 201,634 markers (CBC) for failing these standard measures (clustering, heterozygote rate, annotation updates, missingness <5%). One individual from each pair of first- or second-degree relatives (*n* = 34 PROVIDE; *n* = 1 CBC) was removed. We also checked for individual missingness >2% (none in either cohort), principal components outliers (*n* = 4 PROVIDE; *n* = 31 CBC), and heterozygosity outliers with F > 0.3 (*n* = 4 PROVIDE; *n* = 4 CBC). We filtered out variants with minor allele frequency <0.5% (M = 659,171 PROVIDE; M = 751,869 CBC) and those with Hardy-Weinberg equilibrium *P* < 10^−5^ (M = 789 PROVIDE; M = 85 CBC). Following these filtering steps, we had 499 individuals and 699,246 markers (PROVIDE) and 594 individuals and 826,228 markers (CBC). We split the genetic data into individual chromosomes for phasing and imputation. Phasing was done with SHAPEIT v2.r790 (45, 46) and imputation was done with IMPUTE v2.3.2 (47–51), both using 1000 Genomes Project phase 3 data as the reference (52). Phasing and imputation were done for each cohort separately. 10.8 million variants (PROVIDE) and 10.9 million variants (CBC) had an INFO score ≥ 0.7 and were retained for downstream analysis.

After imputation, PLINK (53) was used to recheck relatedness within each study and no pairs of individuals had PI_HAT > 0.2. The same threshold was used to assess relatedness between the cohorts using KING (54) and 1 individual was removed from PROVIDE. Within PROVIDE, 103 individuals were removed for lack of phenotype information (56 had no diarrheal samples tested, 8 did not have diarrheal samples from the first year, and 39 had missing data for Campylobacter, despite the presence of a diarrheal sample). An additional 10 individuals were removed for lack of covariate data (sex, length-for-age Z-score at birth, length-for-age Z-score at 12 months, water treatment practices). CBC was restricted to those from the Mirpur site for whom TaqMan Array Card data were available, resulting in the removal of 424 individuals. An additional 17 individuals were removed for lack of phenotype information (13 did not have diarrheal samples from the first year and 4 had missing data for Campylobacter, despite the presence of a diarrheal sample) and 4 were removed for missing covariate data (sex, length-for-age Z-score at birth, length-for-age Z-score at 12 months, water treatment practices). After these filtering steps, there were 385 individuals from PROVIDE and 149 individuals from CBC. A complete flow chart of quality control steps can be found in Fig. S1.

10.1128/mbio.00556-22.1 FIG S1 Flowchart of quality control procedures. Download FIG S1, PDF file, 0.1 MB. Copyright © 2022 Munday et al. 2022 Munday et al. https://creativecommons.org/licenses/by/4.0/ This content is distributed under the terms of the Creative Commons Attribution 4.0 International license.

To get a set of independent markers (not correlated) we pruned the data within each cohort. We removed one variant from each pair in a 50 bp window that had r^2^ ≥ 0.05, then shifted the window by 10 bp and repeated the process. After pruning there were 100,033 and 101,951 markers in PROVIDE and CBC, respectively. We used these independent markers to calculate the thresholds of significance using a modified Bonferroni correction. All variants (correlated and not correlated) cannot be used because Bonferroni assumes independent tests (55). The corrected *P* values were *P* = 4.99 × 10^−7^ (0.05/100,033) and *P* = 4.90 × 10^−7^ (0.05/101,951) for PROVIDE and CBC, respectively. We used *P* = 5 × 10^−7^ for both cohorts, which is also consistent with the Wellcome Trust threshold (56). We used this pruned data with smartpca (57) to examine the population substructure. Upon visual inspection, no clustering of individuals was observed and none of the top 10 principal components (PCs) were significantly different between cases and controls by ANOVA (*P* > 0.05).

Next, we examined the distribution of environmental risk factors by case status. We used chi-square tests for categorical data and Welch Two-Sample t-tests for continuous data. Variables with significant differences between cases and controls in a single cohort (*P* < 0.05) were then evaluated together in a binomial regression model. Backward elimination regression was utilized in which the variable with the highest *P* value (>0.1) was removed and the model was tried again. We continued this approach until we identified the model with the lowest Akaike information criterion (AIC), representing the highest quality model of those tested. This resulted in a model for PROVIDE that included sex, LAZ at 12 months, water treatment, and the first PC. To allow for the possibility that growth stunting at birth plays a role in susceptibility to infection despite no statistically significant differences between cases and controls, we opted to include LAZ at birth in the final model. The same process conducted in CBC revealed no significant differences between cases and controls for any of the variables tested. For consistency, the same model was applied to both cohorts.

### Association analysis.

We used SNPTEST (47, 49, 56) to run logistic regression on each cohort separately, assuming an additive model of inheritance and using the scoring method to incorporate the probability weights of the imputed genotypes. We included the five covariates listed above (sex, LAZ at birth, LAZ at 12 months, water treatment, and the first principal component). We then filtered out variants with minor allele frequency < 0.05 (M = 3,884,376 for PROVIDE and M = 4,024,045 for CBC). We also removed variants with IMPUTE2 INFO score < 0.7 (M = 4,798 for PROVIDE and M = 14,705 for CBC). The filtered results were combined in an inverse-variance weighted fixed-effects model using META (58). The output from the meta-analysis was filtered for heterogeneity between cohorts using a *P* value threshold of 0.05, resulting in 6,262,114 variants. Conditional analyses were conducted in the same manner, with each cohort run separately and the filtered results combined using META. Conditional analyses included sex, LAZ at birth, LAZ at 12 months, water treatment, PC1, and rs13281104 as covariates in SNPTEST. The results were filtered for MAF ≥ 0.05, INFO ≥ 0.7, and heterogeneity *P* values (*P*_het_) > 0.05.

### Functional assessment.

To investigate the possible functional impact of the highest-scoring variants, we searched the Genotype-Tissue Expression (GTEx) project (59, 60) for each variant that was in linkage disequilibrium with the highest-scoring variant (r^2^ > 0.8) and had a *P* < 5 × 10^−5^ in the meta-analysis. This enabled us to identify variants that impact gene expression. We also used Ensembl’s Variant Effect Predictor (VEP) (61) to identify the potential consequences of each variant. The placement of enhancers was determined from Ensembl (22) and GeneCards (62).

### eQTL analysis.

A subset of the Bangladesh Environmental Enteric Dysfunction (BEED) cohort (63) (Clinical Trials.gov identifier NCT02812615) was used for eQTL analysis. A total of 81 Bangladeshi adults, 38 Bangladeshi children, and 82 American children were included in this analysis. Duodenal biopsy specimens were collected from children aged 12 to 18 months and adults from 18 to 45 years old. Bangladeshi children were either stunted (LAZ <−2) or at risk of stunting (LAZ = −1 to −2). Bangladeshi adults were malnourished (BMI < 18.5) or healthy (BMI >18.5).

### Sample preparation.

Duodenal biopsy specimens were placed in AllProtect (Qiagen) and stored at −80°C until RNA and DNA extraction. DNA and RNA were extracted from duodenal biopsy specimens with AllPrep kit (Qiagen). Extracted RNA was used for RNA sequencing while DNA was used for genotyping.

### RNA sequencing.

Sequencing libraries were generated using the NEBNext Ultra RNA library preparation kit for Illumina (New England Biolabs). Briefly, mRNA was purified from total RNA using poly(T) oligonucleotide-attached magnetic beads. Fragmentation was carried out using divalent cations under elevated temperature in NEBNext first-strand synthesis reaction buffer (5×). Library fragments were purified with the AMPure XP system (Beckman Coulter, Beverly, MA, USA). PCR products were purified (AMPure XP system), and library quality was assessed on the Agilent Bioanalyzer 2100 system. Clustering of index-coded samples was performed on a cBot cluster generation system using the PE (paired-end) cluster kit cBot-HS (Illumina) according to the manufacturer’s instructions.

Samples were sequenced on a MiSeq (Illumina) and 150-bp PE reads were generated. Raw reads were processed through fastp to remove adapter sequences, poly-N sequences, and reads with low quality. Q20, Q30, and GC content of the clean data were calculated, and only high-quality reads were preserved. Paired-end clean reads were aligned to the reference genome Homo sapiens (GRCh37/hg19) using the Spliced Transcripts Alignment to a Reference (STAR) software. FeatureCounts were used to quantify reads mapped to each gene. Read counts were processed using the bioconductor package DESeq2 v1.30.1 in R (version 4.0.5) and normalized using the DESeq algorithm.

### Genotyping.

Samples were genotyped using TaqMan SNP Genotyping, Assay ID C__27061811_10 (Thermofisher) with CFX96 thermocycler (BioRAD). Relative fluorescence was used to determine genotype. Each sample was categorized as having GG, GA, or AA genotypes.

### Statistics.

Normalized RNAseq counts for genes *ARHGEF10*, *CLN8*, *KCNK13*, and *STX8* were compared between genotypes. Two-sample *t*-Tests were used to determine significant differences in counts between genotypes.

### Data availability.

Data are publicly available from the NIH, via dbGAP, phs001478.v1.p1 (Exploration of the Biologic Basis for Underperformance of Oral Polio and Rotavirus Vaccines in Bangladesh) and phs001665.v1.p1 (Field studies of Cryptosporidiosis and Enteropathogens in Bangladesh), or by request.

10.1128/mbio.00556-22.3 FIG S3 PROVIDE PCA plot. Download FIG S3, TIF file, 0.4 MB. Copyright © 2022 Munday et al. 2022 Munday et al. https://creativecommons.org/licenses/by/4.0/ This content is distributed under the terms of the Creative Commons Attribution 4.0 International license.

10.1128/mbio.00556-22.4 FIG S4 CBC PCA plot. Download FIG S4, TIF file, 0.3 MB. Copyright © 2022 Munday et al. 2022 Munday et al. https://creativecommons.org/licenses/by/4.0/ This content is distributed under the terms of the Creative Commons Attribution 4.0 International license.
