# Supplementary material for: Genome-Wide Association Study of Campylobacter-Positive Diarrhea Identifies Genes Involved in Toxin Processing and Inflammatory Response
Source: mBio. 2022 Apr 14;13(3):e00556-22. doi: 10.1128/mbio.00556-22 (PMC9239263; doi:10.1128/mbio.00556-22)
Supplement: FIG S2 [file mbio.00556-22-s0002.pdf]

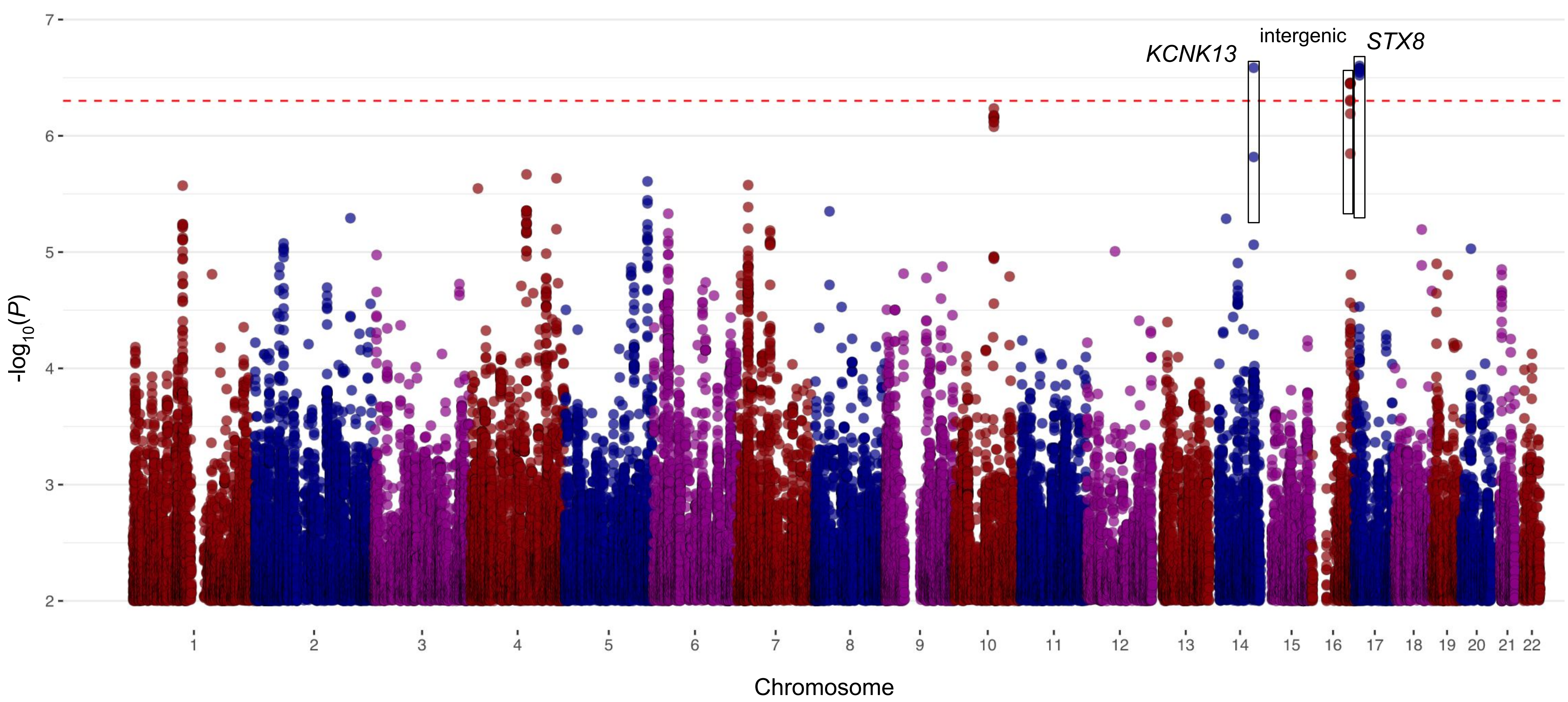

S2 Figure: Manhattan plot of meta-analysis of *Campylobacter*-associated diarrhea in the first year of life, conditioned on rs13281104. Each dot represents a single variant, sorted by chromosomal location along the x-axis. The y-axis is the $-\log_{10}$ *P* value in the meta-analysis of the two cohorts, PROVIDE and CBC. Each cohort was adjusted for sex, LAZ at birth, LAZ at 12 months, water treatment, and the top principal component. The red line indicates genome-wide significance (5×10^{-7}).
