# Supplementary figures and images for: Genome-Wide Association Study of Campylobacter-Positive Diarrhea Identifies Genes Involved in Toxin Processing and Inflammatory Response
Source: mBio. 2022 Apr 14;13(3):e00556-22. doi: 10.1128/mbio.00556-22 (PMC9239263; doi:10.1128/mbio.00556-22)

## PROVIDE

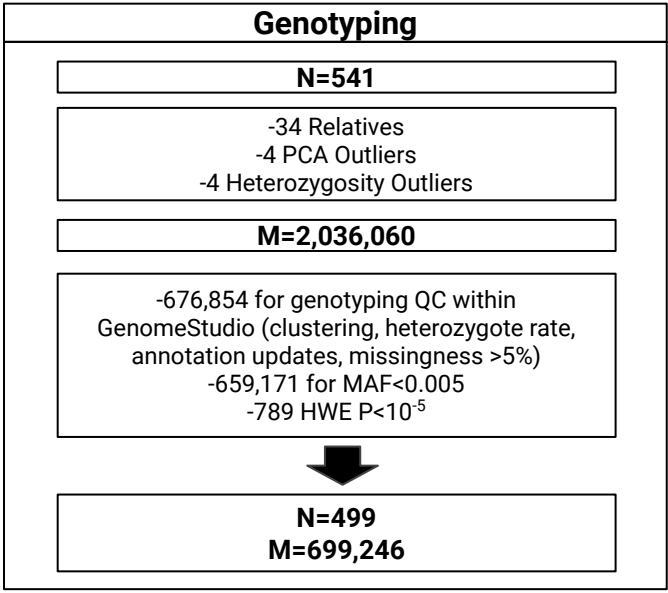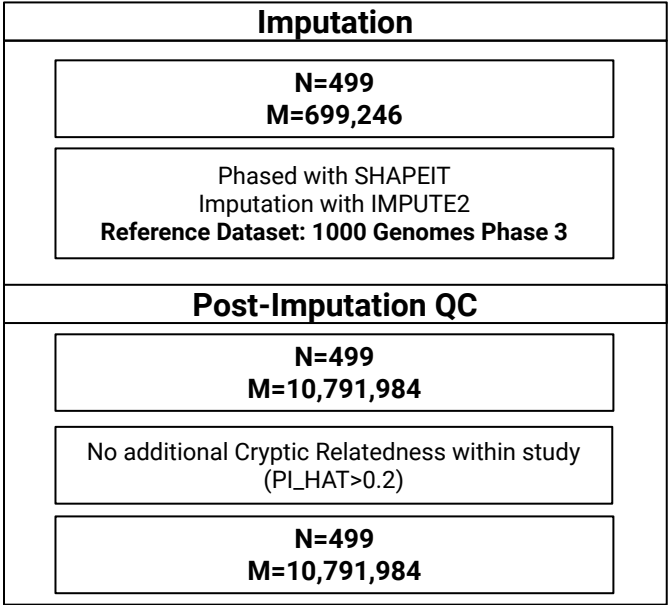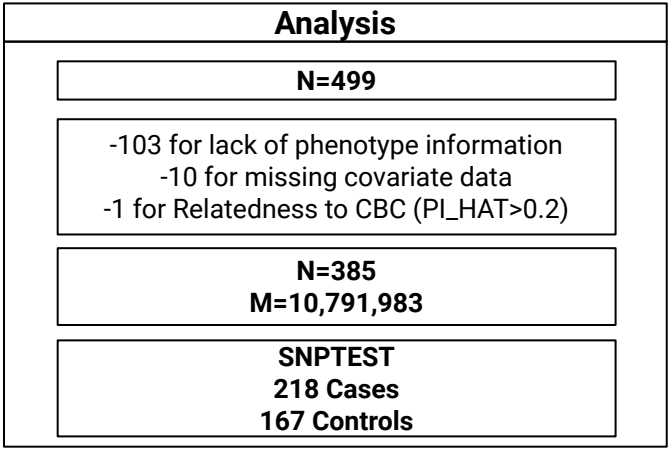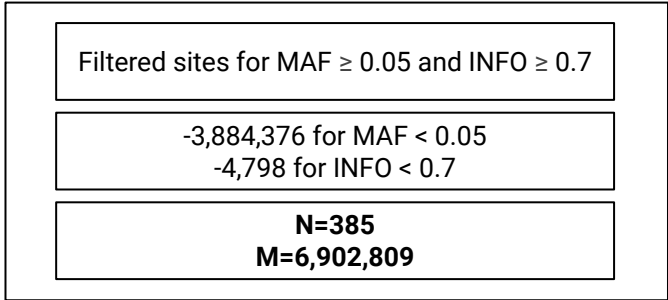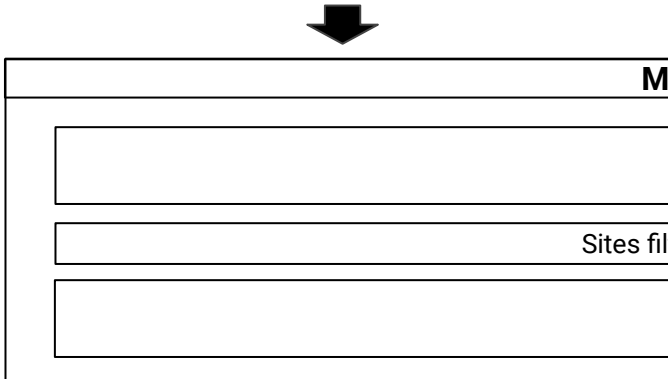

**CBC**

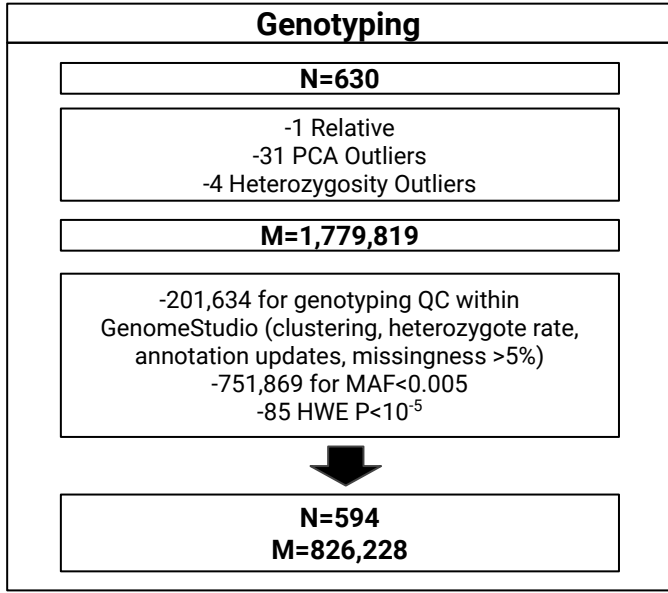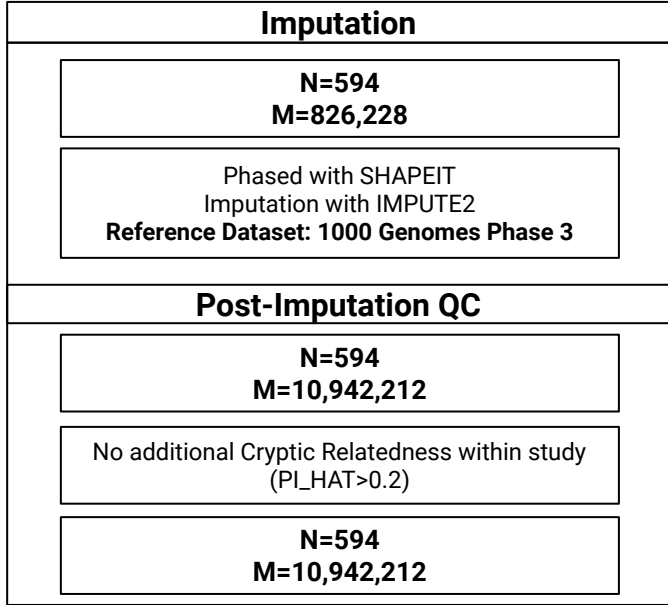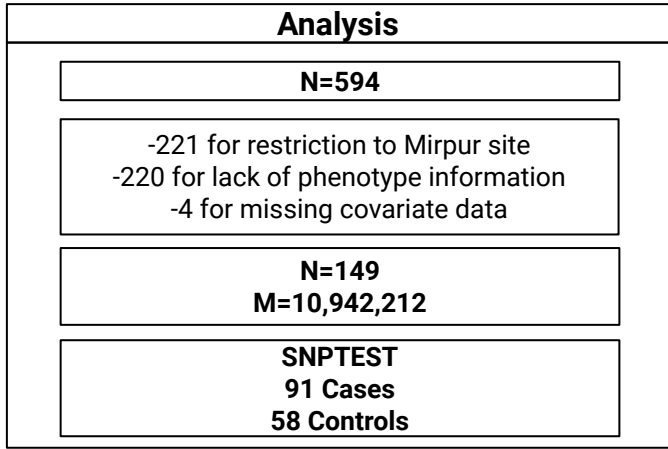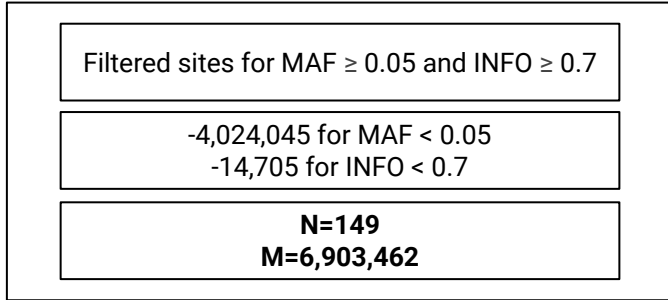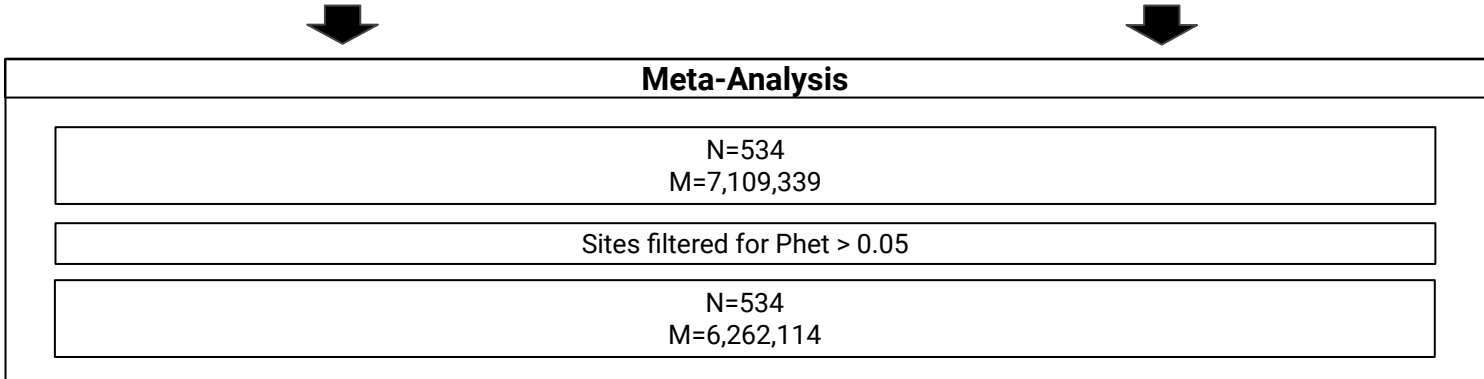

**S1 Figure:** Flowchart of quality control procedures.

Supplement: FIG S1 [file mbio.00556-22-s0001.pdf]

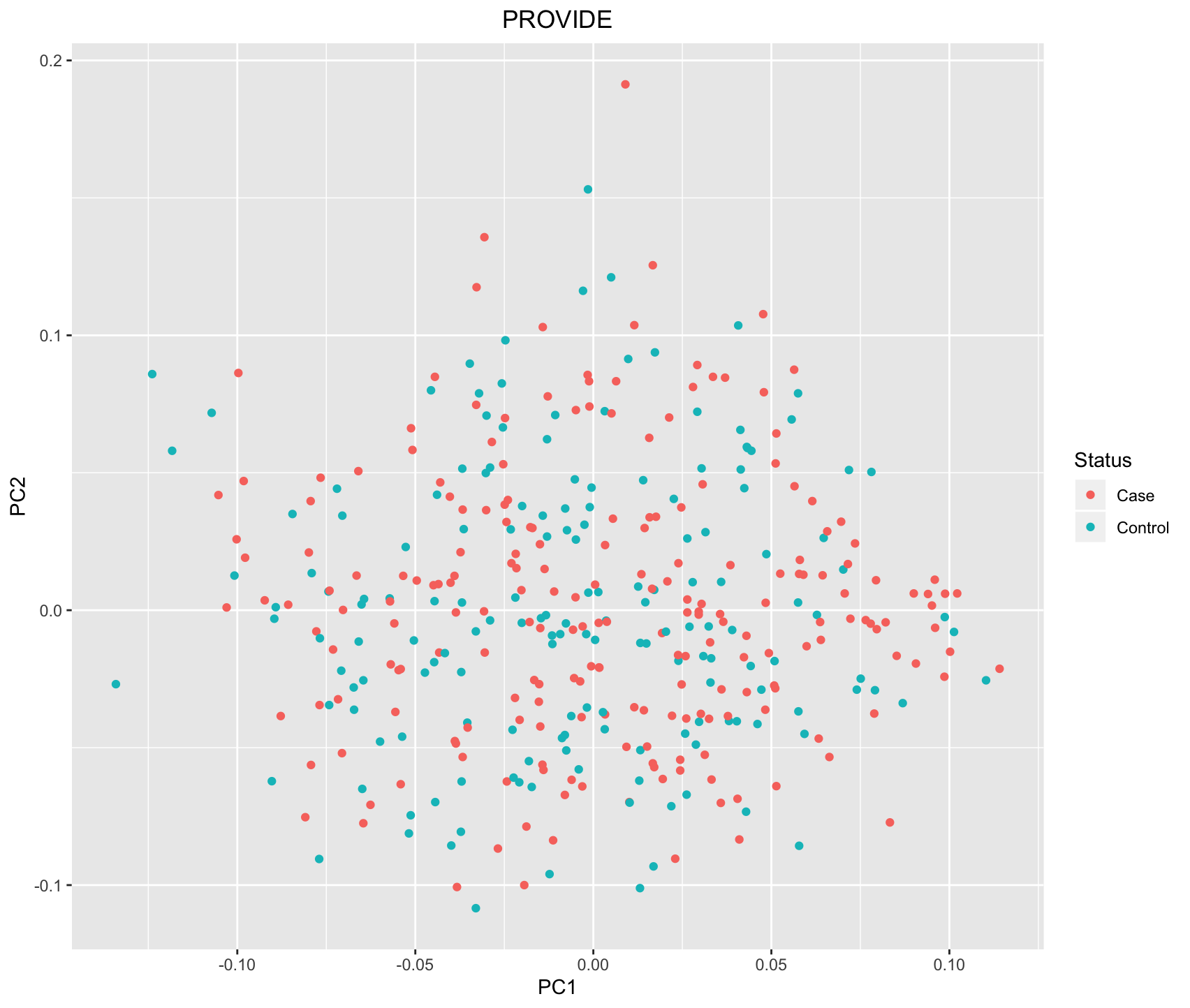

Supplement: FIG S3 [file mbio.00556-22-s0003.tif]

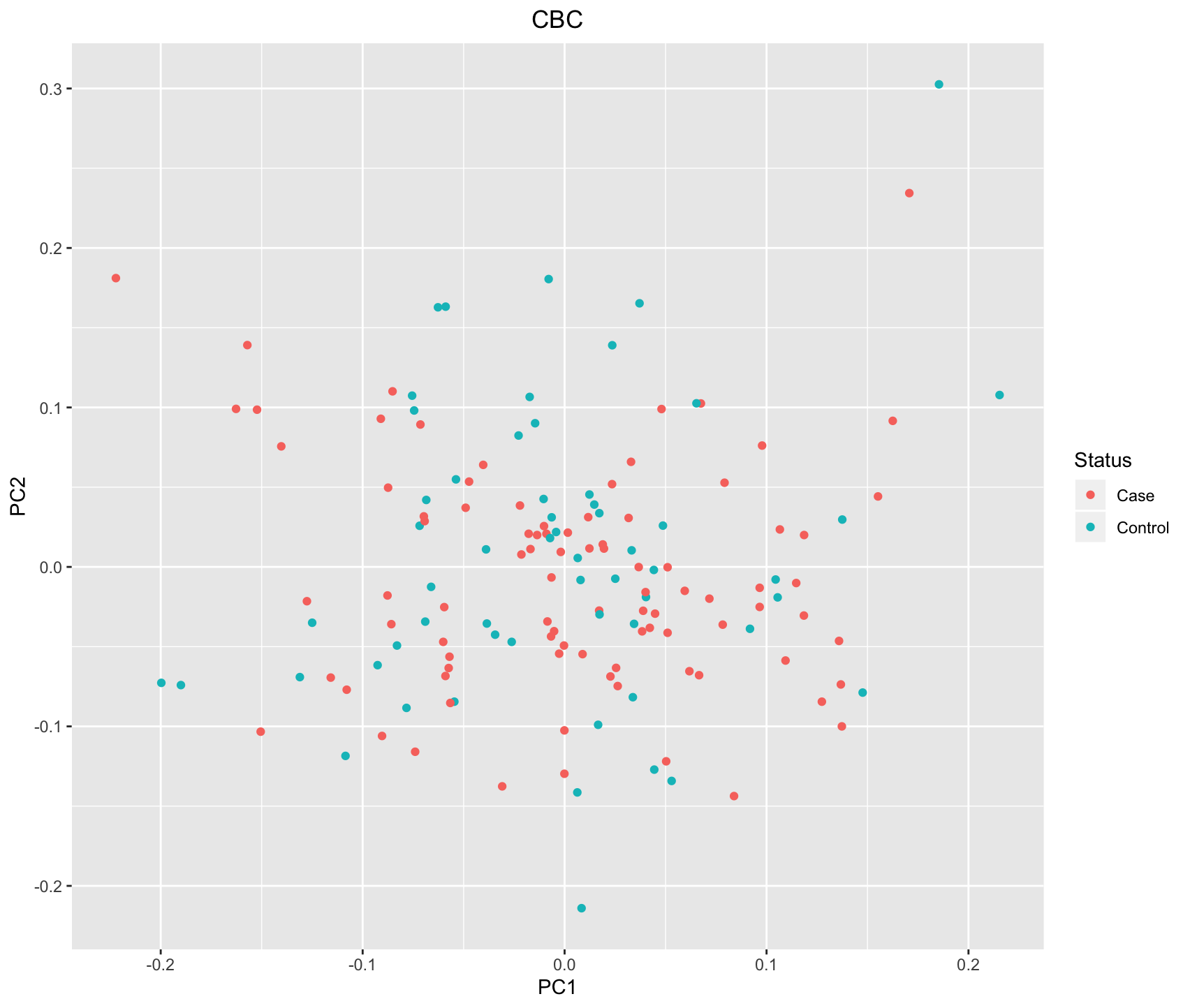

Supplement: FIG S4 [file mbio.00556-22-s0004.tif]
